# Nasal Tip Reconstruction Using Integra Bilayer Wound Matrix: An Alternative to the Forehead Flap

**Published:** 2015-12-02

**Authors:** M. A. Applebaum, J. D. Daggett, W. L. Carter

**Affiliations:** ^a^Division of Plastic Surgery, Department of Surgery, University of South Florida, Tampa; ^b^Plastic Surgery Section, James A. Haley VA Healthcare System, Tampa, Fla

**Keywords:** Integra, nasal reconstruction, bilayer wound matrix, forehead flap, nasal tip

## Abstract

Large defects of the nasal tip with exposure of the underlying cartilage pose a significant reconstructive challenge to the plastic surgeon. **Objective:** This article presents a case of a large nasal tip defect following basal cell excision that was successfully treated with Integra bilayer wound matrix and skin grafting. **Methods:** Following tumor excision, meshed Integra bilayer wound matrix was placed over the exposed nasal tip cartilage. After 4 weeks, the silicone layer was removed and a full-thickness graft placed on the nasal tip. **Results:** This reconstruction resulted in the restoration of patient's nasal tip with good contour and color match without the need for a forehead flap. **Conclusion:** This demonstrates the reconstructive potential of this modality in patients who are not candidates for reconstruction with a forehead flap.

Treatment of large defects of the nasal tip with exposure of the underlying cartilage provides a reconstructive challenge for the plastic surgeon. Skin grafting results in a poor aesthetic outcome with significant contour deformities. Reconstruction of the defect with a forehead flap has been considered a mainstay of treatment and provides an excellent aesthetic result. However, this reconstruction is very demanding from a time and technical standpoint and can be very distressing to patients while in progress. This can limit its application in a patient unable to tolerate long or multiple operations. We present a case report of a large, complex nasal tip defect successfully treated with Integra bilayer wound matrix and skin grafting, with an excellent contour result.

Originally developed by Yannas and Burke in 1980, Integra is made of bovine type I collagen and shark chondroitin-6-sulfate glycosaminoglycan bound to silicone pseudoepidermis.^1^ The bovine dermal collagen analogue allows for host fibroblasts and other cells to be incorporated into the Integra matrix, resulting in the formation of a neodermis. It has been successfully applied to a wide variety of wounds, serving as both a method for promoting vascular ingrowth and providing some bulk to the grafted areas.

Integra has also found use in reconstruction of facial defects. In a separate case, a female patient with basal cell carcinoma (BCC) of the nasal tip appeared to heal by “closed wound healing” via secondary intention. She proved to lack evidence of scarring or contractions after a 6-year period.^2^

## CASE REPORT

**Figure 1:**
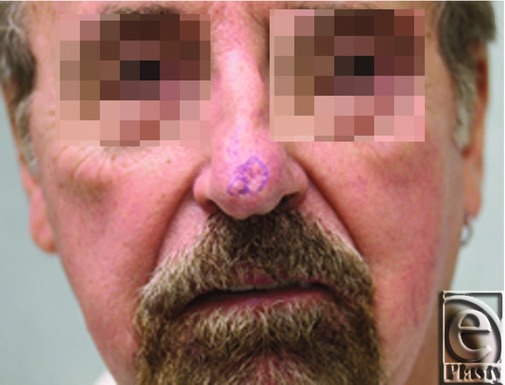
A 68-year-old man with a basal cell carcinoma of his nasal tip.

The patient was a 68-year-old man who presented with a skin lesion of his nasal tip (Fig 1), which was found to be a BCC on biopsy. The patient was recommended to undergo complete excision in the operating room; however, because of a history of multiple comorbidities, including atrial flutter, coronary artery disease, diabetes, cirrhosis, and presence of a pacemaker, he was considered a poor candidate for reconstruction with a forehead flap. Instead, a staged procedure with initial excision and application of Integra bilayer wound matrix was planned to minimize surgical duration.

Following injection with local anesthetic, the BCC was excised with 4-mm margins, resulting in a defect measuring 2 × 2 cm with exposed cartilage in the wound bed. Integra was placed to provide a template for neodermogenesis. It was meshed in a 1:1.5 fashion to allow for adequate contouring to the convex nasal tip.

**Figure 2:**
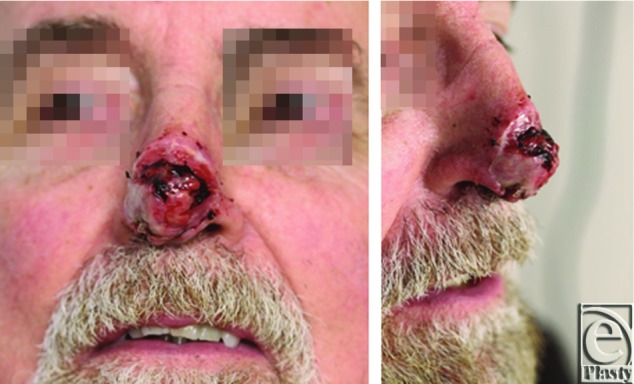
Nasal tip 3 weeks after Integra placement.

Patient was followed on an outpatient basis until there was evidence of sufficient vascularization of the Integra (Fig 2).

**Figure 3:**
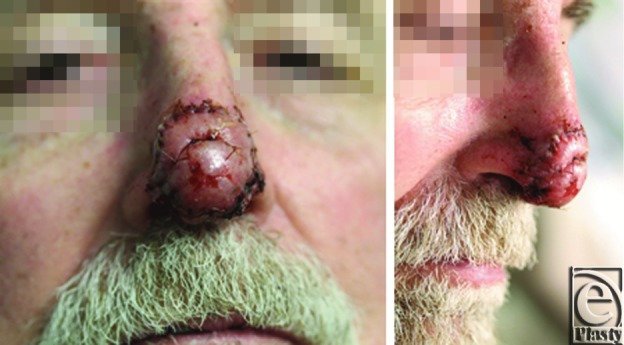
Nasal tip 1 week after full-thickness skin grafting.

Four weeks following his original surgery, he returned to the operating room for the second-stage procedure. The silicone barrier layer was removed, and any areas of pronounced granulation tissue were carefully excised to match the contour of the surrounding wound. A full-thickness skin graft was harvested from the skin of the left neck and secured in place over the defect (Fig 3).

**Figure 4:**
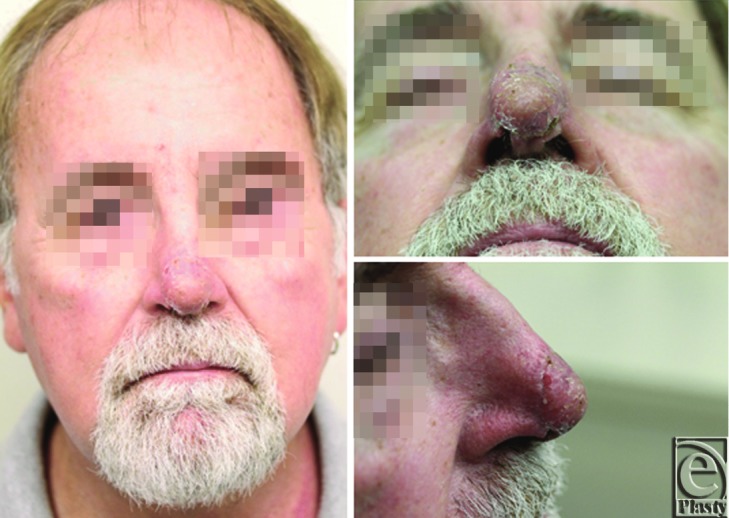
Nasal tip 8 weeks after full-thickness skin grafting.

At 8 weeks postprocedure, the patient appeared to have a minimal pale scar (Fig 4).

## DISCUSSION

There are multiple ways to approach a nasal tip reconstruction. Flaps have traditionally been favored instead of skin grafts, but there are benefits and disadvantages to utilizing both approaches. Aesthetically, flaps have been preferred to grafts because grafts increase the risk of a mismatch of skin tone and contour deformities. In addition, in a situation of exposed cartilage, skin grafting is contraindicated because of the lack of a vascular wound bed. The type of flap procedure that is conducted is based on the size of defect, skin texture, and skin tone. These procedures can be a single transposition flap, a nasolabial transposition flap, and a forehead flap. The larger the defect and closer it is to the nasal tip, the more likely the need for a forehead flap. Once a nasal tip defect reaches 2 cm, a forehead flap is considered to be the gold standard for reconstruction.

In patients with multiple comorbidities, a forehead flap carries a greater healing burden, prolonged operative time, and a higher risk of complication than a graft-based reconstruction. In addition, it requires 2 or 3 stages for completion and, although the end result can be aesthetically pleasing, the interval appearance while the flap remains pedicled can be upsetting and unacceptable to some patients. In such patients, the use of Integra can be advantageous in several respects. By preventing the need for a flap, it minimizes the secondary donor defect and scarring.^2^ In addition, it can fill in volume and contour differences besides allowing for grafting over exposed cartilage. Finally, this reconstruction can easily be performed under local anesthetics

Heimbach et al^3^ also showed that the use of Integra should be the preferred treatment option compared with autograft, allograft, and xenograft with regard to healing time.^4^ They noted faster healing time in patients who had suffered severe burns treated with Integra. The use of Integra on BCC nasal tip reconstruction, however, has not been sufficiently observed but has been shown to allow for shorter healing time and less scarring, in this case, than a forehead skin flap. The healing time, with Integra, was seen to last 8 weeks compared with the traditional forehead skin flap that can be seen to last months.

In summary, Integra bilayer has proven to be a possible alternative to the forehead flap in selected cases that involve large nasal tip defects with exposure of underlying cartilage.
